# Spontaneous Absorption of Extensive Subinternal Limiting Membrane Hemorrhage in Shaken Baby Syndrome 

**DOI:** 10.1155/2014/360829

**Published:** 2014-12-07

**Authors:** Tatiana Tarules Azzi, Leandro Cabral Zacharias, Sérgio Luis Gianotti Pimentel

**Affiliations:** Department of Ophthalmology, University of São Paulo, Clínicas Hospital, 05403-000 São Paulo, SP, Brazil

## Abstract

The Shaken Baby Syndrome (SBS) is characterized by subdural hematomas (SH), retinal hemorrhages (RH), and multiple fractures of long bones without external evidence of head trauma. Subinternal limiting membrane (ILM) hemorrhage, also known as macular schisis, is a characteristic finding of this entity. There is no guideline on the right time to indicate surgical treatment. This report describes an abused child with massive sub-ILM hemorrhage, which showed spontaneous absorption after less than two months of follow-up. Due to the possible spontaneous resolution, we suggest an initial conservative treatment in cases of sub-ILM hemorrhage related to SBS.

## 1. Introduction

The Shaken Baby Syndrome (SBS) is characterized by subdural hematomas (SH), retinal hemorrhages (RH), and multiple fractures of long bones without external evidence of head trauma [1].

The incidence of RH in SBS is of approximately 85% [2, 3]. The RH can present in multiple layers and in different numbers, extending to the periphery. Greenwald et al. [4] was the first author to describe the macular retinoschisis, caused by the accumulation of blood between the inner limiting membrane (ILM) and retinal nerve fiber layer, as highly suggestive of child abuse.

There is no consensus regarding when to proceed to vitrectomy for the clearance of preretinal blood in abused children. However, it is known that a thick vitreous hemorrhage may lead to severe axial myopia and amblyopia if it persists for more than 4 weeks [5].

This report describes an abused child with massive sub-ILM hemorrhage, who showed spontaneous absorption of the blood.

## 2. Case Report

**Figure 1:**
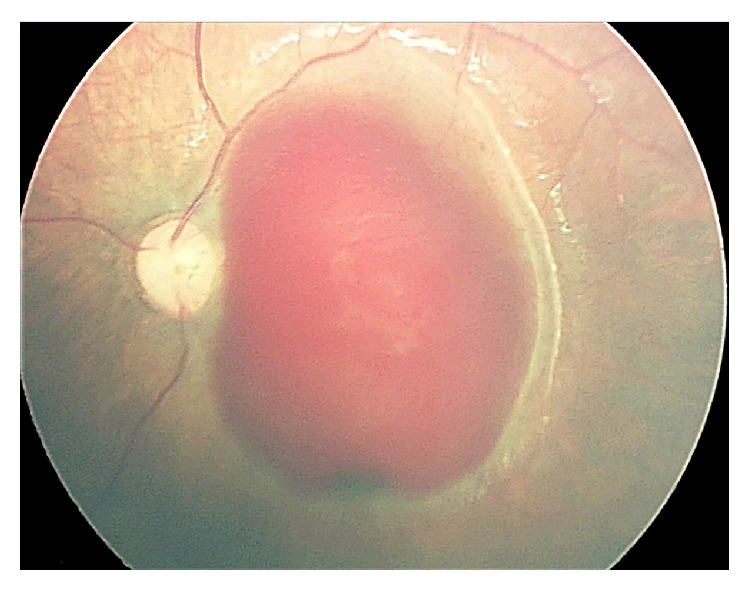
Retinography made by RetCam of the posterior pole of the left eye of an abused child. Note the extensive subinternal limiting membrane hemorrhage.

A 6-month-old male child was referred to our clinic for evaluation after convulsive crisis. The CT scan demonstrated deviation of structures from the median line, presence of subdural hematomas, and obscured cortical sulci. Ophthalmologic examination showed normal pupillary reflexes, symmetric and centered Hirschberg test, and an aggressive reaction to the occlusion of the right eye. On fundus examination, we could note a pale optic nerve at the right eye, and intraretinal hemorrhages were seen in all quadrants of both eyes (OU). A dense, cupuliform, premacular hemorrhage was observed in OU, suggestive of subinternal limiting membrane (ILM) topography (Figure 1). Due to the child's systemic and ophthalmic findings, the hypothesis of child abuse was considered and the social service was notified.

We considered surgery on the left eye of the child, as the right eye had low visual acuity potential related to the disc pallor. However, any ophthalmic surgical intervention at that time was contraindicated by the neurosurgery due to the child's cerebral findings.

**Figure 2:**
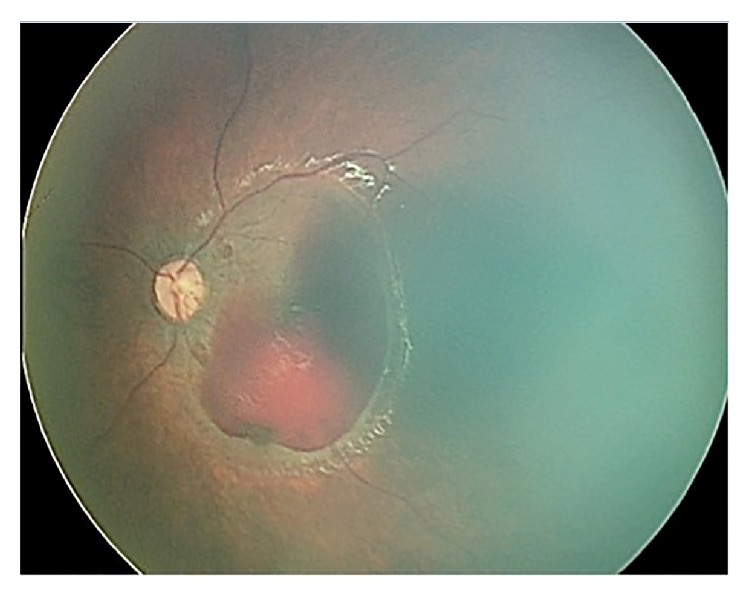
Retinography of the same eye (OS) after 30 days of follow-up. Note the spontaneous decrease in blood volume.

After 30 days of follow-up, there was significant improvement in the sub-ILM hemorrhage in the OS (Figure 2); therefore, the posterior pars plana vitrectomy surgery to drain the sub-ILM hematoma was cancelled.

After 10 weeks of follow-up, both eyes showed spontaneous resolution of the sub-ILM hemorrhage.

## 3. Discussion

The SBS is a form of abusive head trauma, characterized by repeated acceleration-deceleration forces with or without evidence of blunt trauma [6]. In our first contact with the patient, documented by RetCam (Clarity Medical Systems, Pleasanton, CA) extensive sub-ILM hemorrhage compromising both maculas was noticed, and despite the risk of visual deprivation, we had no clearance for surgery, so the child was just followed via serial retinography. To our surprise, there was a rapid and significant improvement in the amount of blood at the macular hemorrhagic retinoschisis even without surgical intervention, with complete resolution in less than 2 months.

Vitreoretinal interventions in children should be conducted with extreme care, as the child eye is very different than the adult: the lens is proportionally thicker, the vitreous cavity is smaller, and the vitreous is denser and more adherent to retinal structures [6]. Therefore, our initial conservative approach can be easily justified by the surgical challenges and risks involved, and as the sub-ILM clearance turned to be faster than expected, we believe a conservative approach may be applied to other similar cases with good functional outcomes.

Despite the blood absorption, we did find a significant visual deficit on the right eye caused by an optic neuropathy, probably related to ischemia or trauma. There is a direct correlation between the severity of HR and the degree of intracranial involvement; children with dense vitreous hemorrhage have poorer visual prognosis, as it often coexists with other retinal and/or brain damage. Cases with intraretinal or subhyaloid hemorrhages tend to have a better visual outcome with less eye and cranial involvement when compared to cases with vitreous hemorrhages [7].

Srinivasan and Kyle described that cases with subhyaloid hemorrhage greater than two disc diameters, dense VH, or diffuse involvement of the fundus were associated with increased acute neurological injury [8], as observed in our report.

Treatment options for traumatic hemorrhagic schisis include observation or vitrectomy. Medele et al. described the use of repeated intravitreal injections of sulfur hexafluoride gas and tissue plasminogen activator (tPA) in four eyes with traumatic schisis, with faster absorption of the hemorrhages [9]. Early surgical intervention is considered to avoid deprivation of visual stimulation (amblyopia) and to decrease axial myopia [9]. There is no consensus on the time of surgery in these cases. Several authors recommend waiting from days to several months [9–11]. In the case reported, it was possible to rule out any surgical intervention after a month of follow-up.

In summary, we report the time for clearance of an extensive sub-ILM hemorrhage in an abused child. Based on our findings, cases of hemorrhagic macular retinoschisis, caused by SBS, could be initially observed and surgery should be considered just if there is persistent hemorrhage involving the visual axis.
